# The impact of Di(2-ethylhexyl) phthalate on human organ development: mechanisms and clinical relevance review

**DOI:** 10.3389/fpubh.2026.1830552

**Published:** 2026-05-04

**Authors:** Xiduo Zhao, Lianyu Hua, Youjun Xiong, Fuyou Shi, Yilin Zhong, Shangwei Zhang, Dongmin Yu, Chengnan Tian

**Affiliations:** 1 The First Clinical College, Gannan Medical University, Ganzhou, Jiangxi, China; 2 Department of Cardiovascular Surgery, The First Affiliated Hospital of Gannan Medical University, Ganzhou, Jiangxi, China; 3 Breast Diagnosis and Treatment Center, The First Affiliated Hospital of Gannan Medical University, Ganzhou, Jiangxi, China

**Keywords:** biological mechanisms, DEHP, endocrine disruption, organ development, public health

## Abstract

Di(2-ethylhexyl) phthalate (DEHP) is a commonly used plasticizer that has raised significant concerns due to its strong endocrine-disrupting effects, which are closely associated with developmental toxicity. While previous reviews have explored DEHP’s developmental toxicity, this study uniquely focuses on recent (past 10 years) advances in understanding the molecular mechanisms behind DEHP-induced organ developmental disorders. It emphasizes key pathways involved in cellular proliferation, differentiation, and endocrine balance. Despite widespread human exposure to DEHP, translating preclinical findings to human health outcomes remains challenging due to variations in exposure levels, individual susceptibility, and limited clinical data. This review compiles the latest clinical research on DEHP-related developmental health risks, explicitly addressing these uncertainties and exposure-related factors. By combining new molecular insights with clinical relevance, this review offers a focused scientific basis for future research into DEHP’s developmental toxicology, bridging the gap between preclinical mechanisms and real-world human exposure outcomes.

## 1 Introduction

Di(2-ethylhexyl) phthalate (DEHP) is a widely used plasticizer that enhances the flexibility and durability of polyvinyl chloride (PVC) and other plastics. It is prevalent in medical devices, food packaging, and various consumer products. Chemically, DEHP features a phthalic acid core esterified with two 2-ethylhexyl side chains, with a molecular formula of C₂₄H₃₈O₄ and a molecular weight of 390.57 g/mol. Its high thermal and chemical stability allows DEHP to resist degradation in diverse environmental conditions. However, its lipophilic nature promotes bioaccumulation in biological tissues, leading to a range of adverse health effects beyond endocrine disruption and reproductive toxicity (1). Research has extensively documented DEHP’s toxic effects across multiple systems, including metabolic disorders like insulin resistance and obesity, immune dysregulation such as impaired immune cell proliferation and cytokine secretion, thyroid function disruption, and gender-specific toxicity. For instance, males may experience more severe reproductive and metabolic impairments, while females may have increased susceptibility to endocrine disorders (2–5). DEHP’s environmental persistence and bioaccumulative potential make it a common contaminant in water and soil systems (6). As a prototypical environmental endocrine disruptor (EED), DEHP disrupts hormonal signaling in humans and animals, causing endocrine imbalances, reproductive dysfunction, developmental anomalies, and other multi-system pathological changes (7).

Global concern over DEHP exposure and environmental contamination is increasing, especially among vulnerable groups like pregnant women and children, where developmental exposure can lead to severe health issues (8). The primary exposure routes include dietary ingestion, dermal absorption, and inhalation, with significant leaching from plastic containers into food and water (9). Bioaccumulation heightens its toxicity during critical developmental periods, potentially causing congenital cardiac defects, endocrine disorders, and other pathological changes. DEHP is mainly metabolized in the liver through hydrolysis by esterases into mono(2-ethylhexyl) phthalate (MEHP), which is further oxidized into secondary metabolites like 5-hydroxy-MEHP and 5-oxo-MEHP (10). These metabolites are mostly excreted in urine; however, both DEHP and its active metabolites can disrupt endocrine balance, leading to reproductive and developmental toxicity (1). Additionally, cytochrome P450 enzymes are crucial in DEHP metabolism, aiding in xenobiotic clearance and the biotransformation of endogenous compounds (11). Metabolite-induced changes in intracellular signaling pathways may further worsen hepatotoxicity and damage to the reproductive system (12).

In real-world settings, humans are typically exposed to complex mixtures of phthalates, bisphenols, and other endocrine disruptors, rather than to DEHP alone. This co-exposure can lead to synergistic or additive toxic effects, making it a crucial consideration in developmental toxicity assessments. To ensure transparency in our literature selection, we outline our criteria for including and prioritizing studies: (1) Human studies, including epidemiological investigations and clinical observations, are prioritized because they directly reflect DEHP’s effects on human health and provide essential evidence for clinical relevance. (2) Animal studies, primarily using rodent models, are included to complement human data, especially when human evidence is scarce. (3) *In vitro* studies, involving cell lines and primary cell cultures, are integrated to elucidate the molecular pathways underlying DEHP-induced toxicity, bridging *in vivo* observations and mechanistic exploration. We assign weight to the evidence based on study rigor, prioritizing high-quality human epidemiological studies and well-replicated animal and *in vitro* studies to support our conclusions. Typical human environmental exposure to DEHP ranges from 0.01 to 0.1 μg/kg body weight/day, which is 3,000–7,500 times lower than doses commonly used in animal and *in vitro* experiments. This significant discrepancy limits the direct translation of experimental findings to human health risk assessments. Most current evidence comes from high-dose animal models and *in vitro* systems, with limited low-dose epidemiological data. It is crucial to note that results from in vitro and animal studies cannot be directly extrapolated to humans due to differences in metabolism, sensitivity, exposure routes, and dose levels. Most experimental doses greatly exceed real-world human exposure, and species-specific responses further limit translational value.

## 2 Literature search strategy

This narrative review utilized a systematic and transparent literature search strategy to ensure comprehensive coverage and minimize selection bias. We retrieved relevant literature published between January 2010 and December 2025 from the PubMed, Web of Science, and Embase databases. The search terms used were: [“Di(2-ethylhexyl) phthalate” OR “DEHP”] AND (“organ development” OR “developmental toxicity” OR “endocrine disruption” OR “mammary gland” OR “cardiac development” OR “liver development” OR “kidney development” OR “brain development”). We included original articles, clinical studies, epidemiological investigations, animal studies, and *in vitro* mechanistic studies that focused on DEHP exposure and its effects on organ developmental toxicity in humans or model organisms. In contrast, we excluded reviews, meta-analyses, conference abstracts, letters, non-English publications, and studies unrelated to developmental toxicity or organ development.

## 3 Effects of DEHP on various human organs

### 3.1 Effects of DEHP on breast development

Breast development begins in the embryonic stage, matures during puberty, and undergoes functional changes during pregnancy and lactation. This process is regulated by endocrine hormones, as well as genetic and environmental factors. With rising environmental pollution, the health risks associated with DEHP exposure are becoming more evident. DEHP can enter the body through diet, inhalation, and skin contact, and it can be transmitted to the fetus via the placenta during pregnancy, potentially affecting fetal breast development (12). However, strong human evidence showing that DEHP alone alters breast development under typical environmental conditions is still limited. Current evidence suggests a possible link between DEHP exposure and abnormal breast development, as well as an increased risk of breast cancer (13). This association should be interpreted with caution, as humans are often exposed to other plasticizers, such as di-n-butyl phthalate (DBP), which may also impact breast development. During embryonic development, breast formation begins with the development of mammary buds. This is followed by significant proliferation and differentiation of breast tissue under estrogen stimulation during puberty. Pubertal breast development is influenced by various factors, including genetic susceptibility, nutritional status, and environmental exposure (14). During pregnancy, breast tissue undergoes significant structural and functional changes, with hormonal shifts promoting the development of mammary alveoli and milk synthesis (15).

#### 3.1.1 DEHP in animal models and human breast studies

DEHP may cause abnormal breast tissue growth by disrupting estrogen and progesterone signaling pathways. However, most evidence supporting this comes from experimental models or cancer-related studies, rather than real-world developmental exposure scenarios. In female Sprague–Dawley rat models, prolonged exposure to DEHP at 150 mg/kg body weight per day resulted in mammary gland hyperplasia and increased ductal epithelial cell proliferation (1). This dosage is significantly higher than internationally recognized tolerable exposure limits. For instance, the U. S. Environmental Protection Agency (EPA) recommends a reference dose (RfD) of 20 μg/kg body weight per day. The 150 mg/kg/day dose is approximately 3,000 to 7,500 times higher than typical human environmental exposure levels, which usually range from 0.01 to 0.1 μg/kg body weight per day. Similarly, high concentrations of DEHP exposure, such as 10,000 nM, significantly increased the proliferation of T-47D breast cancer cells, activated the progesterone receptor (PR) signaling pathway, and altered hormone sensitivity, affecting breast development and pathological states (1). However, these *in vitro* concentrations are also much higher than those found in human biological samples under environmental exposure conditions. As such, they do not accurately represent real human exposure and cannot be directly extrapolated to human breast development.

Epidemiological studies indicate a possible link between DEHP exposure, abnormal breast development, and increased breast cancer risk. However, these findings are inconclusive and require further validation. Exposure to DEHP during pregnancy may lead to structural changes in the breast tissue of offspring during puberty, potentially raising future breast cancer risk. This association, however, is complicated by other environmental and genetic factors. DEHP can also be transmitted to infants through breast milk, impacting breast development (12). Early postnatal exposure routes, such as plastic baby bottles, toys, and medical devices, have been largely overlooked despite their relevance during critical developmental periods like infancy and early childhood. These exposure routes may contribute to DEHP accumulation in the body, affecting breast development since infants and young children experience higher exposure levels relative to their body weight and have immature metabolic systems. Notably, inconsistencies exist between rodent and human data; high-dose effects observed in rodents are not replicated in human epidemiological studies.

#### 3.1.2 Potential mechanisms of breast impact

**Figure 1:**
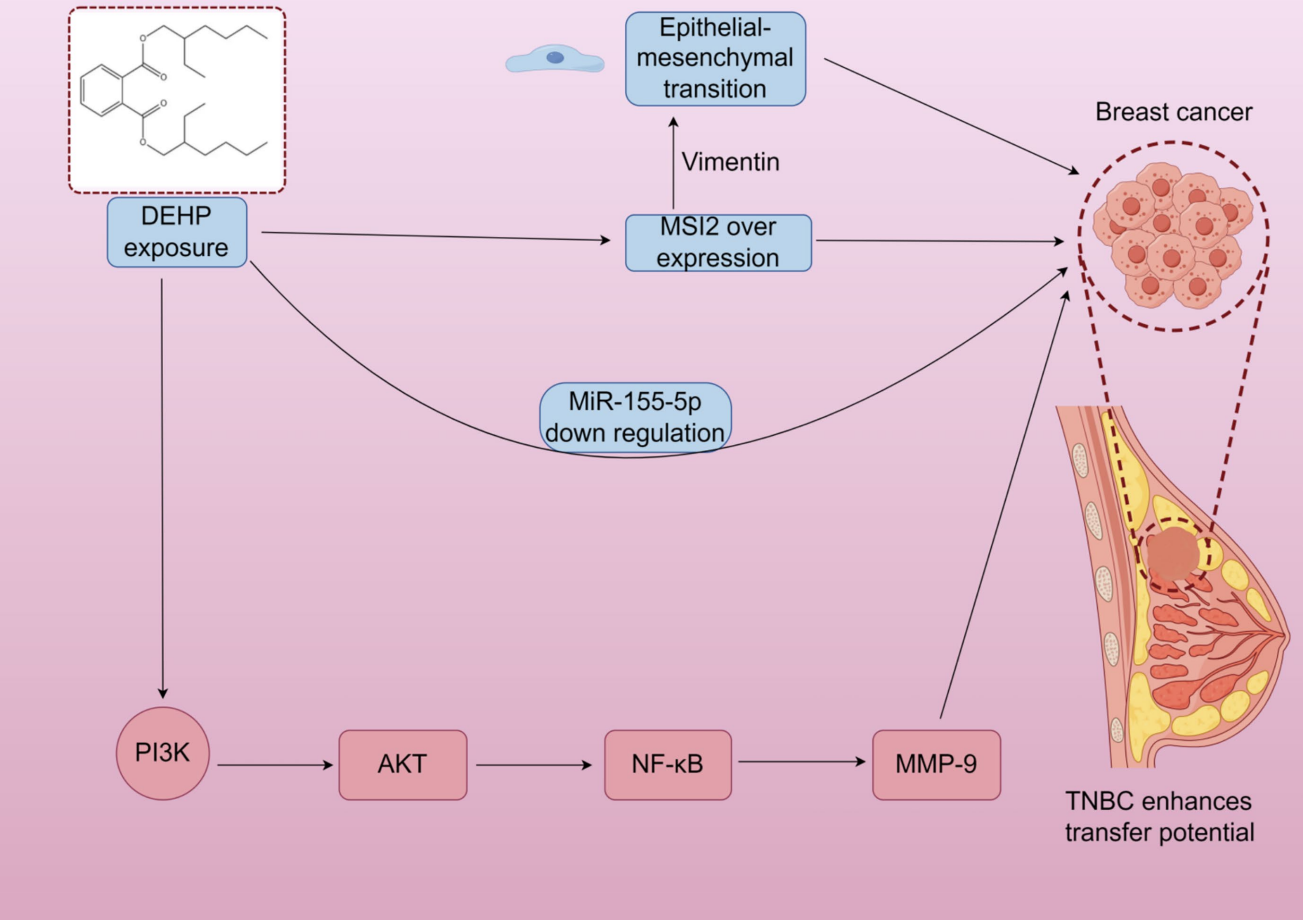
Triple negative breast cancer (TNBC) is a highly invasive subtype of breast cancer. DEHP exposure can induce msi2 overexpression, promote epithelial mesenchymal transition, activate PI3K/Akt/NF - *κ* B/MMP-9 signal axis, and enhance tumor metastasis potential. In addition, mir-155-5p level was negatively correlated with msi2 expression after DEHP exposure.

DEHP may increase the expression of estrogen receptors (ER) and progesterone receptors (PR), activate the PR signaling pathway, and promote breast cell proliferation (10). However, these mechanisms are primarily based on *in vitro* and animal studies, and their relevance to human breast development under environmental exposure remains uncertain. DEHP also activates the PI3K/AKT, MAPK/p38, and TGF-*β*/SMAD signaling pathways. It promotes breast cancer cell proliferation and survival via the PI3K/AKT pathway while inhibiting apoptosis-related proteins, enhancing cell survival (12). Additionally, DEHP may promote tumorigenesis by upregulating inflammatory factors in the NF-κB signaling pathway (16). It is important to note that these findings are largely derived from cancer cell lines or high-dose animal models, limiting their applicability to normal breast development under typical exposure conditions. Triple-negative breast cancer (TNBC) is a highly aggressive breast cancer subtype, with metastasis being the leading cause of mortality. Research indicates that DEHP exposure can induce overexpression of Musashi RNA-binding protein 2 (MSI2), which promotes epithelial-mesenchymal transition through interaction with vimentin. This activates the PI3K/Akt/NF-κB/MMP-9 signaling axis, enhancing tumor metastatic potential (17). After DEHP exposure, miR-155-5p levels decrease. In simulated miR-155-5p treatments, DEHP inhibited TNBC migration, reducing MSI2 and vimentin expression, suggesting a negative correlation between miR-155-5p levels and MSI2 expression. Again, these findings are specific to cancer models and may not directly reflect DEHP’s effects on normal breast development (Figure 1).

### 3.2 Effects of DEHP on cardiac development

The heart is a crucial organ for sustaining life, undergoing a complex developmental process from embryogenesis to maturity. Cardiovascular diseases (CVD) are a leading global mortality cause, affecting millions annually, including congenital heart disease (CHD) in infants (18, 19). Cardiac development begins during the embryonic period, typically completing between the third and eighth weeks of gestation. During this critical phase, the heart’s structural and functional components form, such as atrial and ventricular septation, valvulogenesis, and the cardiac conduction system (20). Exposure to environmental endocrine disruptors like Di(2-ethylhexyl) phthalate (DEHP) during this period can disrupt normal cardiogenesis, potentially leading to congenital heart defects or impaired cardiac function. Cardiac morphogenesis involves the precise regulation of multiple signaling pathways and transcription factors, with the Wnt signaling pathway playing a key role in cardiomyocyte proliferation, differentiation, and apoptosis (21). Neural crest cells are also essential, contributing to the formation of structural elements and functional maturation of the heart (22). Additionally, evidence shows that DEHP induces cardiovascular disorders at various exposure levels. A recent study by Naija et al. (72) confirmed that DEHP exposure disrupts cardiac function and morphology, with dose-dependent effects on cardiomyocyte proliferation and apoptosis (19). Importantly, cardiovascular outcomes often result from combined exposure to multiple plasticizers and environmental pollutants, not just DEHP alone.

#### 3.2.1 DEHP in animal models and human cardiac studies

In murine models, maternal exposure to DEHP during pregnancy has been linked to increased offspring malformations, reduced fetal weight, and shorter crown-rump length. This exposure downregulates genes involved in aerobic respiration and mitochondrial ATP synthesis, indicating that DEHP impairs cardiac function through mitochondrial dysfunction. Specifically, DEHP exposure reduces mitochondrial respiratory capacity in cardiomyocytes, decreases ATP production, and raises reactive oxygen species (ROS) levels (22). In rat models, oral DEHP administration results in decreased heart weight and size, reduced cardiomyocyte proliferation and survival, increased apoptosis, and ventricular septal defects. Mechanistic studies show that DEHP induces cardiotoxicity by suppressing neuregulin-1 (NRG1) expression and inhibiting the ErbB2/ErbB4-PI3K/AKT signaling pathway; however, exogenous NRG1 supplementation can partially reverse these apoptotic effects (23). Zebrafish embryos exposed to DEHP display bradycardia and pericardial edema. Cross-species comparisons reveal consistent patterns of cardiac developmental abnormalities between zebrafish and mice (24). Notably, human environmental exposure to DEHP typically ranges from 0.01 to 0.1 μg/kg/day, whereas animal studies often use doses 100–10,000 times higher, limiting clinical translation, and cardiac responses vary significantly among rodents, zebrafish, and humans, highlighting the need to emphasize translational limitations.

Epidemiological evidence indicates a link between DEHP exposure and increased cardiovascular disease (CVD) incidence. Studies in Asia and Europe have shown a positive correlation between high DEHP exposure and CVD among the older population (25). Women have higher urinary DEHP metabolite levels than men, suggesting possible gender differences in CVD susceptibility (26). In studies on congenital heart disease (CHD), maternal DEHP exposure is associated with a higher risk of neonatal cardiac anomalies (27). Prenatal DEHP exposure is also linked to adverse pregnancy outcomes, such as fetal growth restriction and preterm birth (28, 29). Maternal urinary DEHP metabolite levels during mid-to-late pregnancy are positively related to fetal weight, head circumference, and birth weight, indicating potential long-term effects on fetal development (30). However, animal studies often use high doses, while human studies are observational and may be confounded by other exposures.

#### 3.2.2 Mechanisms of DEHP’S impact on cardiac development

DEHP may impair cardiac development by disrupting hormone signal transduction. However, the changes in gene expression induced by DEHP are highly dependent on exposure dose, timing, duration, and the experimental model system used. There are inconsistencies across studies: some report downregulation of key genes involved in fetal cardiac development, such as *NRG1* and *HIF-1α*, following DEHP exposure, which can lead to ventricular septal defects and asymmetric cardiac structures (22). DEHP exposure significantly increases reactive oxygen species (ROS) levels in cardiomyocytes, leading to oxidative stress, cellular damage, and apoptosis. Research has shown that DEHP exposure is closely associated with mitochondrial dysfunction, reduced ATP synthesis, and ROS accumulation, thereby compromising cardiac development (22). Additionally, DEHP promotes cardiomyocyte apoptosis and exacerbates cardiac developmental abnormalities through the activation of oxidative stress-related signaling pathways. DEHP also alters the expression of genes critical for cardiac development, including the downregulation of cardiac-specific transcription factors *TBX5* and *MEF2*, and the upregulation of *GATA4*. This mechanism may involve changes in DNA methylation status, thereby modulating the transcriptional activity of genes essential for cardiac development (24). However, these findings are not universal, and variability across studies should be acknowledged to avoid overgeneralization. Most mechanistic data lack validation at low doses and are derived from animal or *in vitro* models rather than human cardiomyocytes. Consequently, direct extrapolation to human heart development remains unreliable.

### 3.3 Effects of DEHP on liver development

The liver is a crucial metabolic organ responsible for essential functions such as nutrient metabolism, detoxification, and biomolecule synthesis. In early development, hepatic progenitor cells emerge from the ventral foregut endoderm, progressing through various differentiation stages to become mature hepatocytes and bile duct epithelial cells. This development relies on both intrinsic signaling pathways and extrinsic factors like nutritional status and growth factor signaling (19). Key transcription factors, including FOXA1–3, HNF4α, and GATA4, are vital for regulating gene expression during liver organogenesis (22). The liver also plays a significant role in drug and toxin metabolism, aiding in their elimination and protecting the body from harmful substances (7). Thus, proper liver development and function are essential for maintaining systemic homeostasis, and disruptions can lead to severe metabolic disorders and diseases. Additionally, the liver’s ability to metabolize and transform xenobiotics is crucial for clearing toxicants and maintaining physiological balance (7). Notably, the current section relies primarily on animal studies and high-dose experimental research, with limited understanding of DEHP effects under real-world human exposure levels, which is a key limitation that needs to be acknowledged.

#### 3.3.1 DEHP in animal models and human liver studies

Animal studies have shown that DEHP exposure leads to liver inflammation and fibrosis (31). Maternal DEHP exposure during pregnancy is linked to metabolic disturbances in adult offspring, marked by increased hepatic fatty acid oxidation products and oxidative stress-related metabolites, with more pronounced effects in female mice (32). DEHP exposure is associated with microvesicular steatosis, hepatocyte swelling, and inflammatory responses, indicating hepatotoxicity through disrupted hepatic metabolic pathways (32). Long-term exposure is strongly associated with hepatocarcinogenesis in animal models, suggesting potential carcinogenicity. Rats exposed to DEHP during gestation and lactation show hepatic lipid accumulation and fibrosis in adulthood. Even low-dose exposure can upregulate proteins involved in lipid metabolism, leading to hepatocellular inflammation and histopathological changes (33). DEHP induces hepatocyte apoptosis and inflammatory responses by activating the NF-κB signaling pathway, worsening liver injury (31). Offspring of dams exposed to DEHP during pregnancy and lactation exhibit increased hepatic fatty acid oxidation intermediates and oxidative stress-related metabolites, along with microvesicular steatosis and hepatocyte swelling (32). Importantly, hepatic responses in rodents differ significantly from humans in metabolism and sensitivity, so animal results cannot be directly extrapolated to human liver development.

In adults, prolonged exposure to high doses of DEHP is linked to an increased risk of liver cancer and fibrosis, primarily through oxidative stress and inflammation-induced liver damage (32). Most human studies on DEHP and liver disease are observational and subject to significant confounding factors, such as obesity, dietary habits, and socioeconomic status. In individuals with obesity or diabetes, DEHP can exacerbate liver disease by impairing insulin sensitivity and disrupting lipid metabolism. DEHP and its main metabolites—mono-(2-ethyl-5-hydroxyhexyl) phthalate (MEHHP) and mono-(2-ethyl-5-oxohexyl) phthalate (MEOHP)—are strongly associated with non-alcoholic fatty liver disease (NAFLD) and hepatic fibrosis. Despite the observational nature of most studies and the presence of confounding factors, the link between DEHP exposure and NAFLD remains credible. Elevated urinary levels of DEHP metabolites are positively correlated with serum markers of liver function, such as alanine aminotransferase (ALT) and aspartate aminotransferase (AST), suggesting that DEHP contributes to liver pathology by disrupting lipid homeostasis and promoting pro-inflammatory responses in the liver (34). Furthermore, DEHP affects hepatic gene expression related to lipid synthesis and oxidation, leading to intracellular lipid accumulation and liver cell dysfunction (35). Additionally, humans are often exposed to mixtures of phthalates and other environmental contaminants, which may have synergistic effects on liver injury. Notably, human studies on NAFLD show inconsistent results; some find no association after adjusting for obesity, and low-dose findings in animals are not consistently replicated in humans.

#### 3.3.2 Mechanisms of DEHP’S impact on liver development

**Figure 2:**
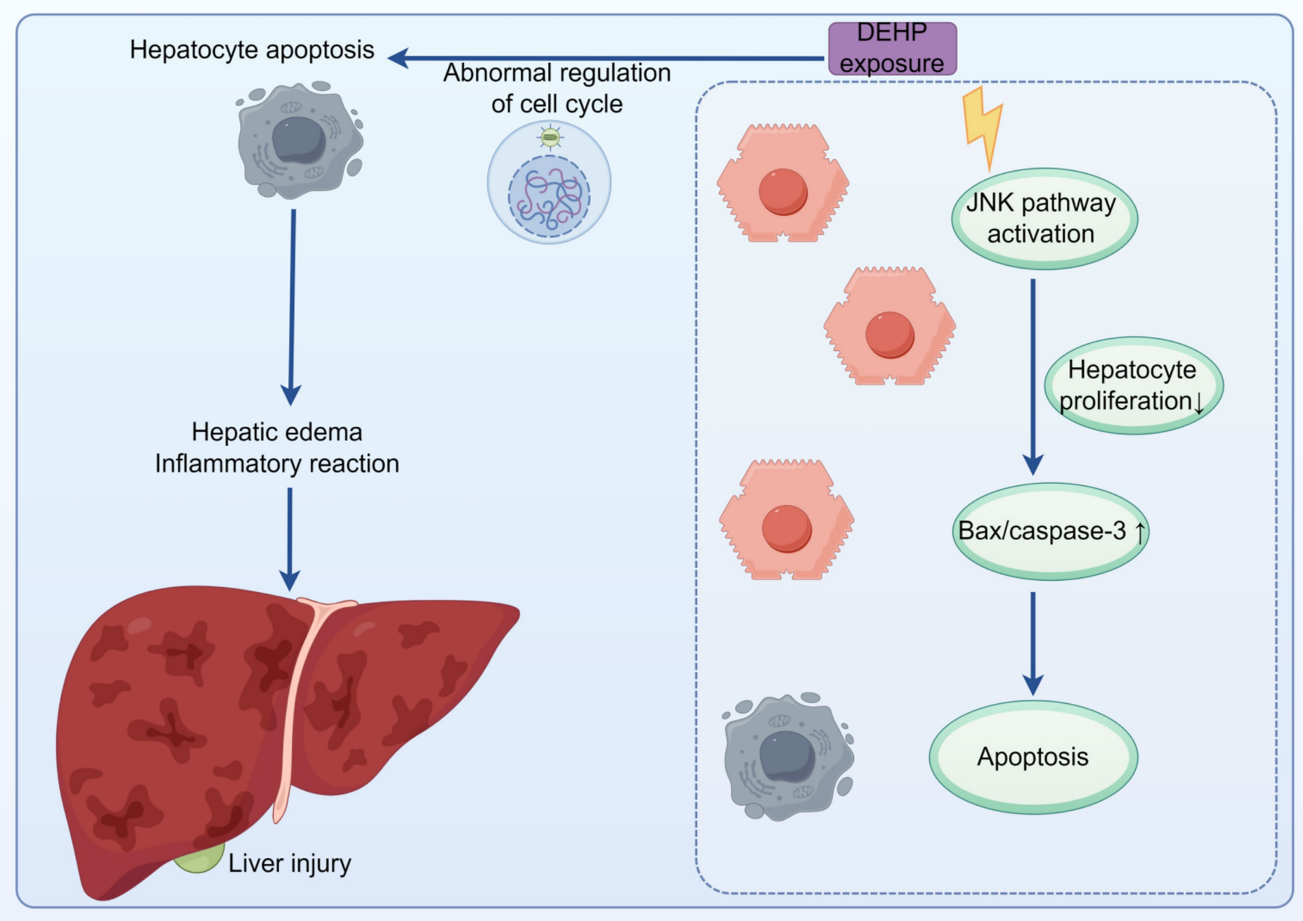
After DEHP exposure, the expression of Bax and caspase was increased by activating JNK signaling pathway, leading to cell apoptosis In addition, DEHP exposure caused abnormal cell cycle regulation and hepatocyte apoptosis, resulting in cell swelling and liver injury.

DEHP triggers oxidative stress, disrupts lipid metabolism, and interferes with endocrine signaling, primarily through its active metabolite, MEHP. Among the mechanisms identified, MEHP’s activation of the PPARα pathway is the most consistently supported by evidence from animal and *in vitro* studies. This pathway influences lipid synthesis and metabolic regulation, directly contributing to hepatic steatosis (36). Additionally, DEHP exposure promotes hepatic inflammation and tissue damage by increasing pro-inflammatory cytokines and decreasing anti-inflammatory mediators (37). DEHP inhibits hepatocyte proliferation and induces apoptosis through the activation of the JNK signaling pathway. This activation increases the expression of pro-apoptotic proteins Bax and Caspase-3 (37). Additionally, DEHP enhances hepatocyte apoptosis by altering the expression of cell cycle regulatory proteins, which leads to cellular swelling and inflammatory reactions (32). Although these apoptotic mechanisms are supported by evidence, they are context-dependent and vary across different exposure conditions. Furthermore, DEHP upregulates genes involved in fatty acid synthesis, such as through increased SREBP-1c expression, contributing to hepatic steatosis (37). This effect aligns with the PPARα-mediated disruption of lipid metabolism, as supported by multiple studies (Figure 2).

Epigenetic modifications, such as DNA methylation and histone acetylation, may play a crucial role in DEHP-induced changes in gene expression. In the liver, DEHP-induced DNA methylation differs from that in other tissues like adipose tissue or reproductive organs. Emerging evidence suggests that some DEHP-induced epigenetic changes, such as transient histone acetylation, can be reversed after exposure stops, especially during early liver development before epigenetic programming is fully established. However, some changes, like abnormal DNA methylation in genes critical for hepatocyte differentiation, may be irreversible, potentially leading to long-term liver dysfunction. Current epigenetic data are preliminary and lack longitudinal validation. All mechanistic insights are derived from rodent or *in vitro* models, and their relevance to human liver development is yet to be confirmed.

### 3.4 Effects of DEHP on kidney development

Kidney development is a sequential and dynamic process that progresses through the pronephros, mesonephros, and metanephros stages, with the metanephros ultimately forming the permanent kidneys. This process is highly coordinated, involving cellular events such as proliferation, differentiation, and tissue remodeling. Kidney organogenesis begins in the fourth week of embryogenesis, marked by the formation of renal tubules and glomeruli, which are the kidney’s fundamental structural and functional units. A critical aspect of this development is nephrogenesis, where nephrons—composed of renal tubules and glomeruli—are formed through the proliferation and differentiation of renal progenitor cells. This process is tightly regulated by key signaling pathways, including Wnt, Notch, and transforming growth factor-β (TGF-β), which control cell proliferation, migration, and differentiation to ensure proper morphogenesis of renal structures and maintenance of renal function (38). Renal tubules are responsible for urine formation and electrolyte reabsorption, while glomeruli filter metabolic waste products from the bloodstream. These physiological processes are precisely modulated by both endogenous factors, such as growth factors, and exogenous influences, like environmental endocrine disruptors. Disruptions in these regulatory mechanisms can lead to abnormal kidney development, resulting in impaired renal function and adverse health outcomes (7).

#### 3.4.1 DEHP in animal models and human kidney studies

Exposure to DEHP can cause significant changes in renal tubules and glomeruli. In quail models, administering 500 mg/kg of DEHP daily resulted in swelling of renal tubular epithelial cells and structural damage to glomeruli (39). This dosage is about 50,000 times greater than typical human environmental exposure levels of 0.01–0.1 μg/kg/day. Additionally, DEHP has been found to trigger inflammatory responses and promote renal fibrosis, which undermines kidney structure and function (40). Animal studies indicate that DEHP exposure raises blood urea nitrogen (BUN) and serum creatinine levels, both markers of reduced renal function. Mice exposed to DEHP show decreased tubular reabsorptive capacity and electrolyte imbalances (41, 42). The nephrotoxic effects of DEHP are linked to the activation of oxidative stress and pro-inflammatory pathways, potentially worsening pre-existing renal injuries, especially in diabetic conditions (43). This suggests that DEHP primarily accelerates the progression of adult kidney disease rather than causing developmental nephrotoxicity. Chronic DEHP exposure may lead to lasting structural and functional kidney impairments, heightening the risk of chronic kidney disease (CKD). Urinary concentrations of DEHP metabolites have been inversely correlated with kidney function markers, indicating that DEHP might accelerate kidney disease progression by disrupting tubular and glomerular integrity (44). However, it is important to note that avian and rodent renal structures differ significantly from those of humans, limiting the translational value of animal-based nephrotoxicity findings.

A study analyzing urine samples from Korean patients with chronic kidney disease (CKD) collected between 2011 and 2020 found an inverse relationship between urinary DEHP metabolite levels and estimated glomerular filtration rate (eGFR) in individuals with moderate renal insufficiency (45). Similarly, data from the U. S. National Health and Nutrition Examination Survey (NHANES) show a significant negative correlation between DEHP metabolite concentrations and kidney function, as well as an elevated urine albumin-to-creatinine ratio (ACR), suggesting that DEHP may negatively impact renal homeostasis (46). Many epidemiological studies on DEHP exposure, CKD, and reduced eGFR are cross-sectional. While these studies highlight an association, their design limits the ability to establish a causal link between DEHP exposure and kidney function decline. Long-term DEHP exposure is associated with structural and functional renal deficits, increasing susceptibility to kidney disorders. Additionally, DEHP exposure may worsen diabetes-related kidney damage by inducing oxidative stress and inflammation (43). This primarily relates to the progression of adult kidney disease rather than developmental nephrotoxicity.

Pregnant women and children are particularly vulnerable to DEHP exposure. Prenatal exposure to DEHP can disrupt fetal kidney development, a condition known as developmental nephrotoxicity. Postnatal exposure in children can impair ongoing renal maturation. Maternal urinary DEHP metabolite levels have been linked to increased risks of fetal growth restriction and preterm birth (28), which may indirectly impact fetal kidney development. In children, DEHP exposure is associated with a higher risk of renal dysfunction, with epidemiological evidence indicating a significant link between DEHP exposure and pediatric kidney diseases (47). Preterm infants, in particular, are at high risk due to exposure to DEHP through medical devices like plastic tubing, ventilators, and intravenous catheters. These infants have immature renal systems and are subjected to high DEHP exposure from medical interventions, making them highly susceptible to DEHP-induced developmental nephrotoxicity, potentially leading to long-term renal dysfunction in adulthood. However, human studies show mixed results regarding eGFR; several large cohorts report no independent effect of DEHP.

#### 3.4.2 Mechanisms of DEHP’S impact on kidney development

DEHP disrupts sex and thyroid hormone levels, impacting renal tubular development during kidney organogenesis and postnatal maturation. This occurs through the activation of estrogen receptors and other endocrine pathways, potentially causing kidney dysplasia or dysfunction (48). Additionally, DEHP may interfere with insulin and growth factor signaling, hindering kidney growth and maturation during postnatal development (49). DEHP activates the p53, MAPK, and Wnt/β-catenin signaling pathways. Activation of the p53 pathway leads to renal tubular cell apoptosis and dysfunction, impacting both developmental nephrotoxicity by disrupting nephrogenesis during organogenesis and worsening existing adult kidney diseases. Aberrant activation of the Wnt/β-catenin pathway is linked to renal tubular dysplasia and reduced kidney function, affecting kidney organogenesis and postnatal maturation (50). Additionally, DEHP can initiate immune and inflammatory responses by activating pro-inflammatory signaling pathways, leading to the release of tumor necrosis factor-*α* (TNF-α) and interleukin-6 (IL-6). This mechanism exacerbates existing kidney diseases in adults and may disrupt normal kidney development during organogenesis and postnatal maturation (51). Chronic inflammation can cause structural damage to renal tubules and glomeruli, alter the local immune microenvironment, impair renal regenerative and repair capacity, and ultimately contribute to the progression of kidney disease.

Evidence indicates that DEHP-induced nephrotoxicity varies with dosage. At low doses, DEHP may disrupt signaling pathways crucial for renal development, while high doses can lead to cell apoptosis and tissue damage. Reversibility of these effects is dose-dependent; mild developmental impairments, such as tubular dysplasia, might be partially reversible if DEHP exposure stops early in development. In contrast, persistent or high-dose exposure could cause irreversible kidney damage. Notably, the mechanisms at low doses are not well-validated, as most studies have focused on animal renal cells, leaving their relevance to developing human kidneys uncertain.

### 3.5 Effects of DEHP on brain development

Brain development is a crucial physiological process in early life, significantly affecting cognitive function and behavior. This complex process involves biological mechanisms such as neural stem cell proliferation, differentiation, neuronal migration, synaptogenesis, and synaptic pruning. Both genetic factors and environmental influences, like nutritional status and exposure to endocrine-disrupting chemicals, regulate these events. Notably, brain development is particularly vulnerable during specific critical exposure periods: the prenatal window (gestation), neonatal window (0–28 days post-birth), and adolescent window (puberty). Each period has unique neurodevelopmental characteristics, and exposure to DEHP during these times can have distinct adverse effects on the nervous system. DEHP exposure impacts brain development through several mechanisms, including oxidative stress induction, gene expression modulation, and disruption of neurodevelopmental signaling pathways. Research indicates that DEHP can induce neuronal apoptosis in the murine brain and hinder neuronal growth and differentiation by suppressing nerve growth factor expression. Furthermore, DEHP may indirectly affect brain development and function by interfering with the endocrine system (52). These findings suggest that DEHP’s neurotoxic effects are closely linked to its negative impact on brain development, with the severity and nature of effects varying depending on the exposure window.

#### 3.5.1 DEHP in animal models and human brain studies

In Sprague–Dawley rat models, exposure to low or high doses of DEHP, during a critical period of rapid synaptic pruning and cognitive maturation, led to structural changes in pyramidal neurons in the CA1, CA2, and CA3 regions of the hippocampus. The hippocampal subregions have distinct roles: CA1 is crucial for learning and memory consolidation, CA2 is linked to social memory, and CA3 is vital for pattern separation and memory encoding. Zebrafish studies show that DEHP exposure significantly alters behavioral responses and gene expression, including the downregulation of the neurodevelopment-related gene *PER3*, indicating potential neurotoxicity (52). Mice exposed to DEHP perform poorly in the Morris water maze test, suggesting deficits in learning and memory (53). The underlying mechanisms include inhibited neuronal survival and synaptic function in the hippocampus, alongside downregulation of the Akt/mTOR signaling pathway (54). Additionally, DEHP increases oxidative stress and inflammatory responses, further impairing neuronal function and contributing to cognitive decline (55). However, neurobehavioral and structural changes observed in rats, mice, and zebrafish cannot be directly extrapolated to human brain development due to significant neurobiological differences.

Prenatal exposure to DEHP during the gestational period is crucial for neural stem cell proliferation and neuronal migration, and is linked to negative neurodevelopmental outcomes in offspring. Specifically, higher urinary levels of DEHP metabolites in pregnant women correlate with poorer cognitive, language, and motor development in their children (52). Socioeconomic factors significantly modify this association by affecting both DEHP exposure levels and the offspring’s vulnerability to its neurotoxic effects. Families with lower socioeconomic status (SES), often marked by reduced income and parental education, experience higher prenatal DEHP exposure. This is primarily due to increased consumption of ultra-processed foods, such as hamburgers, French fries, and soda, which are typically packaged in DEHP-containing materials. In terms of behavioral outcomes like autism spectrum disorder (ASD) and attention-deficit/hyperactivity disorder (ADHD), evidence is varied and inconsistent. Some studies suggest that prenatal DEHP exposure is linked to a higher risk of ASD in children, as environmental endocrine disruptors may disrupt neurodevelopmental processes, potentially causing long-term cognitive and behavioral issues (52). Exposure to DEHP during pregnancy may lead to fetal neurodevelopmental abnormalities, potentially increasing the risk of ASD and other neurodevelopmental disorders (56). However, other studies have not confirmed this association, underscoring the inconsistency in current findings. This discrepancy may arise from differences in co-exposure to other neurotoxicants, such as mercury and cadmium, which independently increase ASD risk, as well as variations in socioeconomic conditions among study populations. Nonetheless, this connection requires further research for validation. Possible mechanisms include placental dysfunction and disruption of hormonal signaling pathways, which can negatively impact fetal growth and development (57).

Exposure to DEHP during early childhood, from the neonatal to preschool period—a crucial phase for synaptogenesis and synaptic pruning—can lead to deficits in attention, learning capacity, and emotional regulation (48). During adolescence, a period marked by ongoing brain maturation and gender-specific behavioral development, DEHP exposure has been linked to ADHD symptoms in some epidemiological studies (58). These pathways are crucial in ADHD pathogenesis and may interact with DEHP to exacerbate symptoms. In adolescence, DEHP exposure can affect gender-specific behaviors and cognitive functions, potentially impairing social skills and emotional regulation. Boys may be more prone to attention deficits and behavioral issues, while girls might experience more significant challenges in emotional and social domains (59). However, findings are not consistent across all studies. This inconsistency might be due to varying levels of co-exposure to other neurotoxins, such as lead, mercury, and cadmium, which disrupt dopaminergic and serotonergic neurotransmission. Additionally, DEHP’s interference with sex hormone pathways could adversely affect reproductive system development and health (60).

#### 3.5.2 Mechanisms of DEHP’S impact on brain development

DEHP can activate the p53 pathway, leading to neuronal apoptosis. In mouse models, exposure to high doses of DEHP results in neuronal apoptosis, marked by increased expression of activated caspase-3 (54). Additionally, DEHP amplifies pro-apoptotic effects by downregulating the anti-apoptotic protein Bcl-2, which may jeopardize long-term neuronal survival (56). These apoptotic processes might also be influenced by endocrine disruption, as hormonal imbalances can affect the expression of proteins related to apoptosis. To address the initial limitation of insufficient integration between endocrine disruption and neurodevelopmental processes, we offer a more comprehensive analysis: DEHP-induced endocrine disruption is intricately linked with neurodevelopmental processes, rather than functioning independently. Specifically, DEHP disrupts the synthesis and signaling of sex hormones, such as estrogen and testosterone, as well as thyroid hormones, which are crucial for regulating neurodevelopment. These hormonal imbalances directly impair neuronal proliferation and differentiation during critical exposure periods, inhibit dendritic development, and reduce synaptic density through oxidative stress and inflammatory responses (54). For example, DEHP-induced estrogen deficiency can suppress the expression of nerve growth factors, thereby impairing neuronal growth and survival. Additionally, DEHP disrupts the intracellular mTOR pathway, further affecting neuronal growth and survival (59).

DEHP disrupts hormone synthesis and signal transduction, causing hormonal imbalances that impair neuronal proliferation and differentiation during critical brain development periods. Evidence indicates dose-dependent and model-specific variations across studies. For instance, high-dose DEHP exposure, which exceeds environmentally relevant levels, often results in more severe neuronal apoptosis and synapse reduction (61). In contrast, low-dose exposure may only slightly disrupt signaling pathways without causing significant neuronal damage. Furthermore, findings in mice may not fully apply to rats or humans, and *in vitro* results may not accurately represent *in vivo* physiological conditions.

### 3.6 Molecular framework of DEHP induced developmental toxicity

DEHP causes developmental toxicity across multiple organs through four key pathways. First, it induces oxidative stress and inflammation by consistently increasing reactive oxygen species (ROS) and activating NF-κB, MAPK, and JNK signaling in the heart, liver, kidney, brain, and mammary gland. This oxidative injury and inflammation lead to apoptosis and tissue damage in these organs. Second, as an endocrine-disrupting chemical, DEHP interferes with estrogen, androgen, progesterone, and thyroid hormone signaling. This hormonal imbalance disrupts cell proliferation, differentiation, and maturation in developing organs. Third, DEHP causes mitochondrial dysfunction by impairing mitochondrial respiration, reducing adenosine triphosphate (ATP) production, and increasing mitochondrial ROS in cardiomyocytes, hepatocytes, and neurons, which collectively drive defective organogenesis. Fourth, DEHP leads to epigenetic reprogramming by altering DNA methylation and histone acetylation in the liver, heart, and brain, resulting in persistent changes in gene expression and long-term developmental defects. Moreover, these pathways interact synergistically. Endocrine disruption exacerbates oxidative stress, mitochondrial damage intensifies epigenetic changes, and inflammation further disrupts hormonal regulation. Together, these interactions form a unified cascade that underpins DEHP-induced multi-organ developmental toxicity.

## 4 Public health impacts and protective measures of DEHP

### 4.1 Epidemiological studies on DEHP exposure and population-level impacts

Typical human exposure to DEHP ranges from 0.01 to 0.1 μg/kg body weight per day, aligning with safety reference doses set by the EPA. However, experimental studies often use doses between 1 and 500 mg/kg per day, representing a 100–50,000-fold increase, which is crucial for interpreting public health implications. DEHP is a widely used plasticizer prevalent in the environment and is known to have several adverse health effects across all age groups and populations. Epidemiological studies consistently link DEHP exposure to chronic diseases such as metabolic syndrome, cardiovascular disorders, reproductive dysfunction, neurodevelopmental disorders, and liver and kidney damage. For example, DEHP’s disruption of sex hormone synthesis and signaling is evidenced by epidemiological findings: urinary DEHP metabolite levels (e.g., MEHP, MEHHP) are inversely related to testosterone and follicle-stimulating hormone (FSH) levels in men, with stronger associations observed in infertile individuals (56). This indicates that DEHP-induced endocrine disruption at the molecular level directly impacts reproductive health disparities at the population level.

DEHP’s role in causing oxidative stress and inflammatory responses is linked to allergic diseases in children and cardiovascular issues in adults (62). Animal studies show that chronic DEHP exposure leads to neurodevelopmental problems, which are supported by epidemiological data. This data connects prenatal DEHP exposure to reduced cognitive function, a higher risk of autism spectrum disorder (ASD) (1), and attention-deficit/hyperactivity disorder (ADHD) symptoms in children. These findings underscore the translation of mechanistic insights into broader population health outcomes. Furthermore, recent epidemiological studies indicate that DEHP exposure may worsen existing health conditions such as diabetes and chronic kidney disease. This is consistent with evidence that DEHP increases oxidative stress and inflammatory responses in specific organs.

Global exposure to DEHP is prevalent, with urinary DEHP metabolite concentrations found in over 90% of the population in industrialized countries like the United States and European Union member states, and 85% in developing nations (63). Over the past decade, biomonitoring trends indicate a gradual decline in urinary DEHP metabolite levels in countries with strict regulatory measures, such as the EU and the U. S., due to decreased DEHP usage in consumer products. For instance, in the U. S., average urinary MEHP concentrations dropped by 35% from 2005 to 2020 following EPA recommendations (64). Conversely, developing countries, including some in Southeast Asia and Africa, show higher exposure rates, with urinary DEHP metabolite levels 2–3 times greater than those in industrialized nations. This is largely due to less stringent regulations, the widespread use of DEHP-containing plastic products like food packaging and medical devices, and enforcement challenges (63). Regional exposure differences are further influenced by industrial activities; areas with intensive plastic manufacturing, waste disposal, and agricultural plastic use, such as certain regions in China and India, exhibit significantly higher environmental DEHP concentrations, resulting in increased population exposure (65). These exposure patterns correlate with regional disparities in DEHP-related health outcomes, with higher rates of reproductive and neurodevelopmental disorders reported in areas with elevated exposure.

Due to the significant public health risks associated with DEHP, various countries have enacted regulations to limit its use. The European Union, for instance, has classified DEHP as a substance of very high concern (SVHC) under the REACH regulation, banning its use in children’s products, medical devices, and food contact materials above 0.1% (66). In the United States, the Environmental Protection Agency (EPA) has conducted risk assessments for DEHP, setting a reference dose (RfD) of 20 μg/kg body weight per day and advising reduced use in consumer products, especially those for children and pregnant women (64). Additionally, countries like Canada, Japan, and South Korea are intensifying their monitoring and regulation of DEHP in plastics, establishing national standards for DEHP levels in food, water, and air to safeguard public health and the environment. Despite these efforts, global regulation of DEHP is inconsistent. Some developing countries struggle with enforcement due to limited monitoring capacity, inadequate regulatory frameworks, and economic pressures to use cheaper DEHP-containing materials, leading to higher exposure levels and increased public health risks (63).

## 5 Future research directions and protective recommendations

Future research should aim to refine methodologies for evaluating the impact of Di(2-ethylhexyl) phthalate (DEHP) on breast development. By integrating big data analytics and machine learning, researchers can more effectively analyze the complex interactions between biomarkers and environmental factors (13, 47). As our understanding of DEHP-induced cardiotoxicity advances, the use of gold nanostars (AuNSs) combined with metal–organic frameworks (MOFs) presents a promising method for ultra-sensitive detection of DEHP in aquatic environments, achieving detection limits as low as 0.75 × 10^−7^ M (67). Developing point-of-use devices with real-time monitoring and removal capabilities could facilitate rapid detection and elimination of DEHP. For understanding DEHP’s effects on liver development, high-throughput screening technologies could efficiently identify potential detoxifying agents. Luteolin, for instance, has shown promising detoxification properties in murine hepatocytes (68). Metabolomic approaches can deepen our understanding of the metabolic disturbances caused by DEHP exposure and help identify key metabolites linked to hepatic injury (32). Regarding DEHP’s impact on kidney health, large-scale longitudinal studies are urgently needed to establish causal links between DEHP exposure and declining renal function. These studies should also evaluate differential susceptibility across populations, including children, pregnant women, and older adults. Developing novel biomarkers for early detection of DEHP-related nephrotoxicity is crucial. To explore DEHP’s effects on brain development, employing animal models and human cell line systems is recommended for systematically investigating underlying mechanisms. High-throughput sequencing and single-cell RNA sequencing technologies can reveal potential molecular pathways, allowing exploration of DEHP’s influence on gene expression and cellular functions (32). Future research should also prioritize translational studies that connect mechanistic findings with population-level health outcomes, especially in vulnerable groups. This approach will inform more targeted public health interventions.

**Figure 3:**
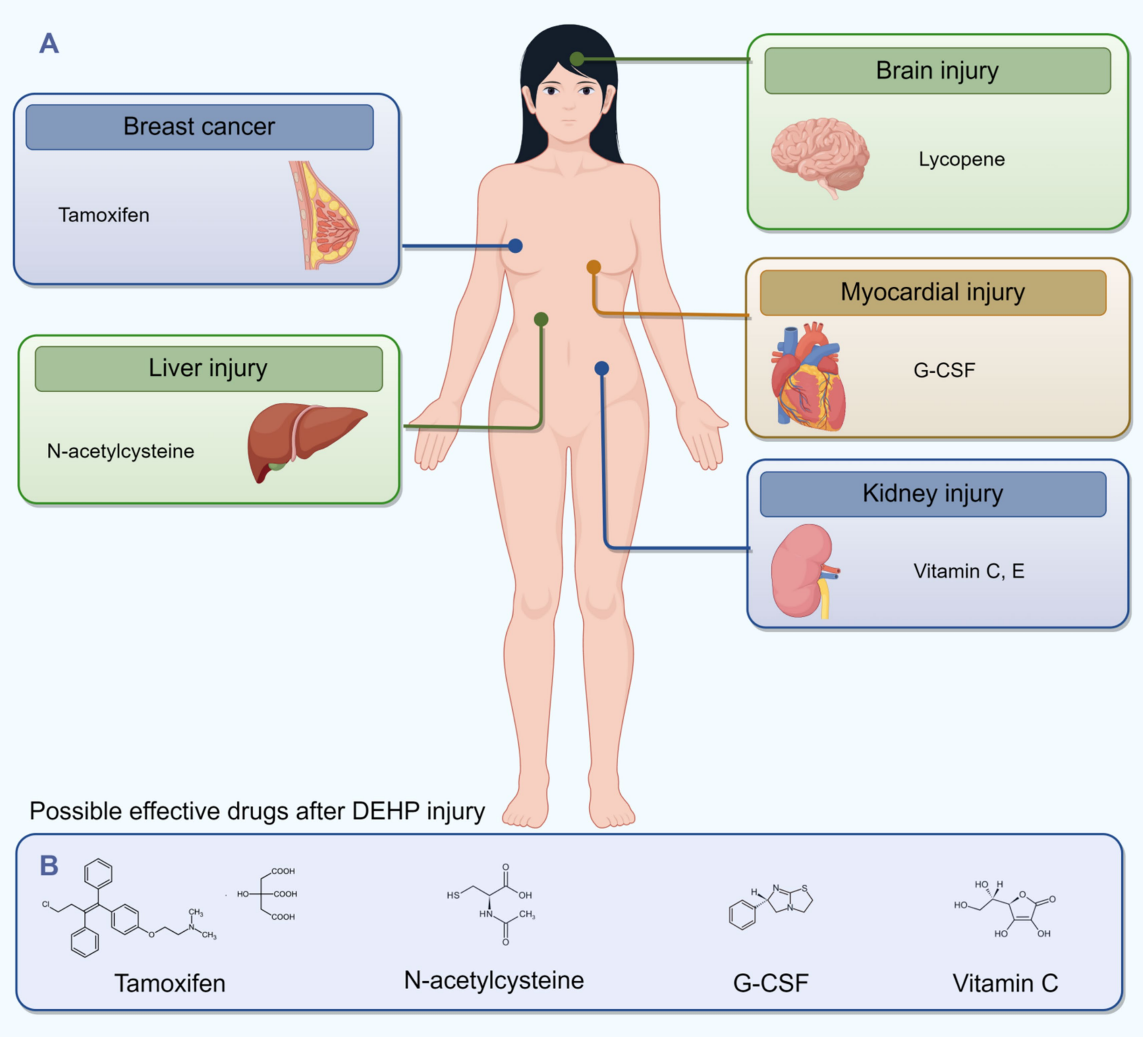
Diagram of a female figure highlighting organs affected by DEHP injury with corresponding potential drugs: tamoxifen for breast cancer, lycopene for brain injury, N-acetylcysteine for liver injury, G-CSF for myocardial injury, and vitamins C and E for kidney injury. Below, chemical structures for tamoxifen, N-acetylcysteine, G-CSF, and vitamin C are presented with their names listed.

To mitigate DEHP’s impact on breast health, individuals should minimize environmental exposure, choose DEHP-free products—especially during pregnancy and lactation—and follow regular breast health exams and early screening protocols. For DEHP-related breast diseases like breast cancer, tamoxifen can inhibit tumor progression by targeting estrogen receptors. In cases of DEHP-induced cardiotoxicity, granulocyte colony-stimulating factor (G-CSF) helps improve myocardial injury by promoting stem cell mobilization and reducing fibrosis and apoptosis, aiding cardiac recovery (69). Antioxidant and anti-inflammatory agents may also reduce DEHP-mediated cardiac damage (70). Preventive measures should focus on reducing exposure to DEHP-containing products during critical developmental periods, such as pregnancy and childhood, to lower long-term cardiovascular risks. Maintaining a balanced diet and regular physical activity can enhance cardiac resilience and decrease cardiovascular events (68). For DEHP-induced liver injury, minimizing exposure and limiting the use of DEHP-containing plastics are advised. Vitamin E and lycopene have shown protective effects against DEHP-induced oxidative stress and liver damage (71), while N-acetylcysteine may improve DEHP-related liver dysfunction (31). In addressing DEHP-associated kidney injury, antioxidants and anti-inflammatory drugs can alleviate oxidative stress and inflammation, preserving renal function (59). Health education, dietary optimization, and increased physical activity are effective strategies for reducing kidney disease risk. To combat DEHP’s neurotoxic effects, promoting DEHP-free alternatives and raising public awareness of exposure risks are crucial. Natural antioxidants like lycopene and vitamin E offer potential neuroprotection against DEHP-induced toxicity. Public health campaigns should also be launched to educate vulnerable groups, such as pregnant women and parents of young children, about sources of DEHP exposure and personal protective measures (Figure 3).

## 6 Conclusion

Research has confirmed that DEHP [Di(2-ethylhexyl) phthalate] exhibits widespread and complex toxicity, impacting various human organ systems, including the mammary glands, cardiovascular system, liver, kidneys, and central nervous system. A comprehensive review of current evidence has identified potential biological mechanisms underlying DEHP toxicity, such as endocrine disruption, gene expression modulation, and apoptosis induction. These insights provide a crucial scientific basis for understanding DEHP’s health impacts and aid in developing informed public health policies. However, most evidence comes from *in vitro* and animal studies with doses 100–50,000 times higher than typical human environmental exposure (0.01–0.1 μg/kg/day), making direct extrapolation to humans inappropriate and significantly limited.

As awareness of DEHP’s harmful effects grows, it is crucial to enhance exposure monitoring, especially among vulnerable groups like children and pregnant women. The variability in study results highlights the need for careful interpretation, considering differences in experimental design, sample characteristics, and outcome measures. To advance understanding, it is essential to harmonize diverse research methods to reach a consensus and to encourage ongoing, rigorous investigation into DEHP’s toxicological profile.

In summary, the implications of DEHP exposure reach beyond scientific research, impacting public health policy and population health outcomes. Strengthened collaboration between researchers and policymakers is essential to enhance exposure monitoring, advance mechanistic and epidemiological research, and implement evidence-based interventions. These efforts are vital for safeguarding public health, reducing preventable risks linked to DEHP, raising awareness about chemical safety, and promoting healthier environments.
